# Recruitment strategies should not be randomly selected: empirically improving recruitment success and diversity in developmental psychology research

**DOI:** 10.3389/fpsyg.2015.00523

**Published:** 2015-04-29

**Authors:** Nicole A. Sugden, Margaret C. Moulson

**Affiliations:** Brain and Early Experiences Laboratory, Department of Psychology, Ryerson University, Toronto, ON, Canada

**Keywords:** recruitment, research methods, diversity, sampling, developmental psychology

## Abstract

Psychological and developmental research have been critiqued for the lack of diversity of research samples. Because differences in culture, race, and ethnicity can influence participant behavior, limited diversity limits the generalizability of the findings. These differences may also impact how participants behave in response to recruitment attempts, which suggests that recruitment itself may be leveraged to increase sample diversity. The goal of the current study was to determine what factors, within a recruitment interaction, could be leveraged to increase success and diversity when recruiting families with children for developmental research. Study 1 found three factors influenced success: (1) recruitment was more successful when other potential participants were also interested (i.e., recruiters were busy), (2) recruiters of particular races were more successful than recruiters of other races, and (3) differences in success were related to what the recruiter said to engage the potential participant (i.e., the script). The latter two factors interacted, suggesting some recruiters were using less optimal scripts. To improve success rates, study 2 randomly assigned scripts to recruiters and encouraged them to recruit more vigorously during busy periods. Study 2 found that two factors influenced success: (1) some scripts were more successful than others and (2) we were more successful at recruiting non-White potential participants than White participants. These two interacted, with some scripts being more successful with White and other scripts being more successful with non-White families. This intervention significantly increased recruitment success rate by 8.1% and the overall number of families recruited by 15.3%. These findings reveal that empirically evaluating and tailoring recruitment efforts based on the most successful strategies is effective in boosting diversity through increased participation of children from non-White families.

## Introduction

It is a recognized problem in many areas of research, including psychology, that the majority of research participants in normative samples have historically been and currently are White (Behl et al., 2001; Miller and Cross, 2006; Yancey et al., 2006). There are many reasons for this over-representation of a single group. One practical reason is that the lion’s share of studies surveyed takes place in countries in which the majority of citizens are White (Henrich et al., 2010). A more theoretical rationale is that many capacities being studied have been assumed to be universal and uninfluenced by the demographic characteristics of the sample of participants (e.g., perception in adults; Henrich et al., 2010). Reflecting this, historically many journals did not require the inclusion of participant demographic details, publishing articles with limited or omitted descriptions of the characteristics of the sample (Yancey et al., 2006). Even if these descriptors were included, some research databases (e.g., Pubmed) used offensive nineteenth century racial categories of questionable relevance and limited utility (e.g., Caucasoid, Sankar, 2003). (For a discussion of issues relating to current categorizations of ethnicity in developmental psychology see Gjerde, 2014.) In response to the ongoing problem of a lack of diversity in research samples, in 1994 the American National Institutes of Health (NIH) formally recognized the ethical duty to be inclusive and a practical need to ensure generalizable research in a multi-cultural world: NIH introduced a requirement that samples include significant numbers of ethnic and racial minorities and of women (National Institutes of Health, 2014).

Psychology has not been immune from homogenous sampling. Six years after the NIH revisions, a review of applied psychology articles found that only 61% reported participant ethnicity (Case and Smith, 2000). In the studies that did report ethnicity, the samples roughly reflected the proportion of White people in the population from which they were drawn, although African Americans were over-represented and Hispanic Americans were under-represented (Case and Smith, 2000). Pediatric psychology research has been found to include few minorities and rarely discuss issues of diversity (Clay et al., 2002). In developmental psychology there has been a push to remedy the issue. In her editorials commencing her tenure as Editor of *Developmental Psychology* and then *Child Development*, Garcia Coll (2005, 2013) notes that it is no longer adequate for contemporary research to focus on White children as normative subjects; samples should be more diverse, inclusive, and representative. In their introduction to a special issue of *Child Development* on race, ethnicity, and culture in child development research, Quintana et al. (2006) argue for the inclusion of more culturally diverse research participants and more culturally diverse models of development. Despite these laudable goals, a recent analysis of sampling in developmental psychology found 41.4% of articles in top developmental journals either failed to report ethnicity or reported it qualitatively (e.g., majority white), making it difficult to discern whether the sample was representative or diverse (Bornstein et al., 2013).

Diversity can either be conceptualized as noise in the data, as was historically the case when the data was not homogenous, or as a central variable that provides deeper understanding of the data (Quintana et al., 2006; Jensen, 2012). Empirical research has found extensive support for the impact of previously underreported participant characteristics, such as culture, race, and ethnicity, on the outcome of a study. Research comparing results from White, Educated, Industrialized, Rich, Democratic (WEIRD) populations with results from non-WEIRD populations have found differences in areas long considered to be culturally invariant or “universal” (Henrich et al., 2010). For example, WEIRD populations are outliers in their basic perceptual skills, cooperation, spatial reasoning, inferential induction, morality, self-concept, motivation, and heritability of IQ (Henrich et al., 2010). Basic emotional responses (Chiao et al., 2008) and self-concept (Chiao et al., 2009) vary across cultures. The differences in WEIRD populations may be visible during infancy and childhood, for example in differing trajectories of motor development compared to non-WEIRD populations (Karasik et al., 2010). These differences may also manifest in differences in the brain (Chiao and Cheon, 2010), leading to a call for neuroscience to consider the impact of culture in research studies (Chiao et al., 2013). Even if not interested in the impact of race, culture, and ethnicity on the phenomenon under study, including these variables can add depth to researchers’ understanding of a phenomenon, provided the sample is adequately diverse to consider these factors (Quintana et al., 2006). Narrowing the diversity of the sample to include only WEIRD participants can limit the depth to which the data can be understood, distort the results, reduce generalizability, and damage overall relevance of an otherwise well-executed study. Given the push to recruit representative samples and the appreciation of the benefits of sample diversity, why do some samples remain homogenous?

There are several challenges to recruitment that may increase the likelihood of recruiting a homogenous sample. Some samples are less diverse than others due to the location in which the research is being conducted: parents constrained by the convenience of accessibility of the lab may be less likely to participate (Sugden et al., 2013). Similarly, geography on a larger scale impacts sampling heterogeneity: conducting research in a WEIRD country, such as Canada, necessarily constrains the diversity of the potential sample in some ways. If a researcher is recruiting a typical sample in Canada (e.g., not visitors or recent immigrants), participants will have had at least some exposure to the Educated, Industrialized, Rich, and Democratic culture that is the latter part of WEIRD. However, given the ethnic diversity of the populations in most large cities which house research institutes and universities (e.g., Statistics Canada, 2007, 2011; U.S. Census Bureau, 2011), a representative sample should not be homogenously White. A sample from the population should include participants that represent the diversity of that population. If the population is not homogenously or nearly-homogenously White, but research samples recruited from the population are nearly ubiquitously White, this disconnect should be probed and remediated.

### Recruitment is Important

The way in which members from the population become members of the sample is through their recruitment into the study. If the sample does not reflect the population, then researchers are somehow recruiting a narrow range of people to participate in their studies. Recruitment strategies will determine who is contacted and the response rate (i.e., proportion of people who agree as compared to those who do not agree to participate), thereby determining how likely it is that the sample is representative of the population from which it is drawn or how likely it is to systematically exclude certain types of people (Patel et al., 2003; Kline, 2009; Bornstein et al., 2013). Adequate sampling requires going beyond a sample of convenience, which violates the basic assumptions of random sampling and typically does not result in a representative sample (Quintana et al., 2006). Unfortunately, the most common type of sampling in developmental psychology is convenience sampling, which is unlikely to be generalizable, representative, or diverse (Bornstein et al., 2013).

Most researchers are aware of the need to be inclusive and view it as important (Taylor, 2008). A minority have voiced concerns about their ability to successfully include minorities in their studies due to perceived challenges in recruiting a diverse sample (e.g., Marshall, 1994; Buist and Greenlick, 1995). Across multiple research areas and populations of interest, there is evidence to support the position that some groups are less likely to participate in research. A review of recruitment research found that the adults who are least likely to volunteer to participate in psychiatric research are those who are older, male, of non-White race, have low educational attainment, and/or are unemployed or of low socio-economic status (Patel et al., 2003). A more recent review, however, found equivocal and contradictory evidence of decreased probability of participation in health research based on ethnicity or race (Yancey et al., 2006). The difference in recruitment success may lie with the study topic, recruiters, or recruitment strategies.

Researchers who view recruitment of a diverse sample as challenging are more likely to use recruitment strategies that are less effective in recruitment of a heterogeneous sample, while researchers who view sample diversity as important are more likely to use inclusive strategies (e.g., recruiting adults for clinical trials, Swanson and Ward, 1995; Williams and Corbie-Smith, 2006). This suggests that the failure to engage lies with the researcher, not with any group of potential participants. This does not mean that researchers are intentionally recruiting ineffectively or excluding non-White participants. A parsimonious and fair explanation of the data suggests two non-mutually-exclusive reasons why researchers are (potentially inadvertently) recruiting a homogenous sample: (1) a lack of diversity in the people being asked to participate, and (2) use of strategies that are differentially effective in recruiting White and non-White participants.

Ensuring that both White and non-White participants are being asked to participate is *sine qua non* of sample diversity. Since participants are unlikely to seek out researchers, it is incumbent on the researcher to seek out participants. There is some evidence that non-White participants may not be asked to participate at the same rate as White participants. A sample of African American adult participants identified one barrier to research participation that speaks to the issue of recruitment strategies: They do not participate in research simply because they had never been asked to participate (Hatchett et al., 2000). When asked to participate, they were “eager to speak out on relevant issues” (Hatchett et al., 2000, p. 671). Hatchett et al. (2000) suggest this reflects a systemic failure to adequately seek them out. A second study, examining whether trust issues were at the root of African American adults’ presumed unwillingness to participate in health research, did not find higher rates of refusal by African American adults, as compared to the general population (49.1 and 49.6%, respectively; Corbie-Smith et al., 2002). Selecting recruitment locations requires an understanding of the diversity of people within the study catchment. Knowing this, appreciating any variation between who could and who is being engaged may illuminate whether the location is appropriate for the goals of the study.

Once a diverse sample of potential participants is being accessed, researchers should consider how they are being asked to participate. There is a general consensus that how recruiters communicate to the participant is important. The way in which a message is framed and the language used in that communication is known to influence how people perceive and interpret the message (e.g., Nelson et al., 1997; Kellermann, 2007). Small changes in words, framing, or sentence construction can significantly change how people respond to the message and whether people would agree to or with it (e.g., Nelson et al., 1997; Kellermann, 2007). Culturally-sensitive or culturally-specific framing might also improve recruitment efforts (e.g., in adults, Swanson and Ward, 1995; Fletcher and Hunter, 2003). Alternatively, technical, complex, difficult-to-understand information, can contribute to potential adult participants rebuffing recruitment efforts (Swanson and Ward, 1995; Gorelick et al., 1998). There is evidence that considering how the message is framed is also effective in recruiting families for developmental research. For example, when asking parents to enroll their infant into a clinical trial, positive framing that high-lighted the benefits of participation increased the likelihood of potential participants agreeing to participate, particularly those participants who were more directly impacted by the issue (Donovan and Jalleh, 2000). Unfortunately, in adult studies, researchers typically do not invest as much effort into recruitment design as study design (e.g., for psychiatric research, Patel et al., 2003), often fail to consider cultural concerns (e.g., in clinical trials, Williams and Corbie-Smith, 2006), and find it a challenge to use simple, clear, lay vocabulary (e.g., in clinical trials, Stark et al., 2002). This lack of attention to the design and evaluation of recruitment efforts may be contributing to some of the challenges identified; however, this has not been extensively studied and evidence for what is and is not effective when recruiting families with children is sparse.

### Empirical Evaluation of Recruitment is Long Overdue

Given that recruitment is important in determining the make-up of the research sample and the sample determines the generalizability of the findings, it is surprising that recruitment itself is not well researched. Highlighting its importance, several researchers have called for empirical evaluation of recruitment. Swanson and Ward (1995) have identified a need to test specific recruitment strategies to evaluate their potential success rate and potentially which combinations of strategies result in recruitment of an adequately diverse sample. Ashing-Giwa (1999) has called for research into factors that influence non-White adults’ participation or non-participation in health research. This message has been echoed in other fields, including developmental research (e.g., Behl et al., 2001; Bornstein et al., 2013). Understanding what factors impact success when recruiting families with children for developmental psychology research is necessary and overdue.

The main goal of this project was to empirically improve recruitment success and diversity while recruiting a developmental population of families with children. To do this, our first aim was to determine which factors predicted recruitment success within a typical recruitment interaction—that is, what makes parents more or less likely to volunteer their child for research. The second aim was to leverage these factors to recruit a more representative sample of families with children by experimentally manipulating the predictive factors.

## Study 1: Exploratory Evaluation of Factors that Impact Recruitment Success

Our first study evaluated multiple factors of a typical recruitment interaction and, with an exploratory analysis, attempted to determine which were most highly correlated with success. We hypothesized that qualities of the recruiter, such as race, gender, and age, would impact success; we expected that parents would be more likely to sign-up when approached by same-race and female recruiters. We also hypothesized that the script the recruiter used (i.e., what they said when they approached the potential participant) and when the recruiter approached the participant (e.g., if their co-recruiter was busy when the recruiter approached a new potential participant) would correlate with success. With respect to the potential participant, we hypothesized that participant gender, ethnicity, and observable qualities of the group (i.e., the number and characteristics of the people who accompanied the potential participant) would all impact whether the recruiter was successful. In particular, we expected recruiters would be most successful with White potential participants, females, and people in smaller groups.

### Method

This study took place at a typical recruitment event: a Developmental Studies recruitment booth at the 2011 Fall BabyTime Show. The BabyTime Show is a large twice-per-year trade show that caters to parents-to-be, parents of infants, and parents of children and usually draws 30,000 attendees to each 3-day event (BabyTime Show, n.d.).

#### Participants

Thirteen recruiters, five non-White (Hispanic, Jamaican, Pakistani, Persian, and Filipino) and eight White, ranging in age from 20 to 30 years old (Mean = 23.13; SD = 3.11; range: 20–30), attempted to recruit families for future developmental studies. The majority of recruiters had experience recruiting participants at other recruitment events. As was typical for our recruitment events, all recruiters received at least 1 h of intensive training prior to attending the event. As part of their training, they were given suggestions on how to approach participants and exemplars of scripts that had previously been used to engage families. Although some recruiters had a preference for certain scripts or anecdotally felt that some scripts were more successful than others, none of these scripts had been evaluated to determine whether they were differentially successful. Recruiters were informed that they were not restricted to the suggested scripts. Recruiters were not blind to the aims of the study, since they had all provided informed consent in order to participate. This research was approved by the Ryerson University Research Ethics Board.

#### Procedure

As was typical, recruiters attempted to engage potential participants (e.g., parents, parents-to-be, and families) who were attending the event. If they successfully engaged a potential participant, the recruiter described what research participation would entail. If the potential participant was interested, the recruiter collected their contact information so that they could be contacted in the future to arrange participation. Regardless of whether the potential participant provided their contact information, they were offered a small toy or gift. Successful recruitment was defined as the potential participant providing their contact information so that they could be contacted to participate in research studies.

A single observer observed all interactions between recruiters and potential participants for 10 min every hour. The observer was dressed identically to the recruiters, wearing an “Infant Scientist” t-shirt and black pants, and carried a similar clipboard upon which, instead of collecting participant contact information, the observer noted their observations. The observer recorded the recruiter involved in the recruitment attempt, the script (i.e., what the recruiter first said to the participant), whether the other recruiter(s) was (were) engaged with other participants during the attempt, characteristics of the targeted potential participant (i.e., gender, ethnicity, pregnancy status, and number and type of people accompanying them), and the outcome (e.g., successful recruitment, refusal). Potential participant ethnicity was simplified to White and non-White, due to known issues in correctly identifying ethnicity. Similarly, pregnancy status was inferred based on observable signs of late pregnancy (e.g., belly protrusion) or self-reported pregnancy. If the participant offered the recruiter a reason for their refusal (e.g., lives in a different city, no children), this was noted.

### Results

Of the 409 recruitment attempts observed, recruiters attempted to recruit men 13.7% of the time (*n* = 56) and women 86.3% of the time (*n* = 353). Of the women recruiters attempted to engage, 57.5% (*n* = 203) were not obviously pregnant, while 42.5% (*n* = 150) were obviously pregnant. Since we are targeting a developmental sample of infants and children, pregnant women represent early recruitment of infants. Most potential participants (56.2%, *n* = 230) were with one, 18.6% of participants (*n* = 76) were with two, 5.6% (*n* = 23) were with three, and 1.2% (*n* = 5) were with four or more other person(s). The remaining participants were alone (18.3%, *n* = 75). Of the potential participants, 40.1% (*n* = 164) were non-White and 59.9% were white. The percent of White and non-White participants observed was not significantly different than the 2006 Canadian Census’ proportion of visible minorities in the city population (42.9% visible minority; Statistics Canada, 2007) from which the sample was drawn [*t*(408) = 1.155, *p* = 0.249].

Over the 3-day event, 472 families were recruited, a non-significant increase of four families over the past 2 year average of 468 families per show. Since some families had more than one child, 529 children were recruited. Of the 409 observed recruitment attempts, 32.8% (*n* = 134) were successful, 54.3% were unsuccessful (*n* = 222), and 13% (*n* = 53) were ineligible or did not clearly accept or decline. Ineligible participants were those that reported that they lived too far away, had already agreed to participate, or did not have children. Responses that were not a clear acceptance or rejection were ones where potential participants said they had to get their partner/spouse or where potential participants said that they would return later. Some participants did return, but these returns did not typically occur during the same observation period and consequently their outcome was not necessarily documented. Excluding the ineligible participants, recruitment success rate was 37.6%. Our analysis included only participants who were eligible and clearly accepted or declined (*n* = 356).

The goal of this first study was to determine which factors predict the outcome of a recruitment attempt. We performed a forward binary logistic regression analysis using recruiter ethnicity, potential participant gender, potential participant ethnicity, interaction between recruiter and potential participant ethnicity, whether other recruiters were busy speaking to other parents during the recruitment attempt, interaction between potential participant ethnicity and whether other recruiters were busy, what the recruiter said to the potential participant (script), and an interaction between participant race and script to predict the outcome (success or failure). Prior to entering pregnancy into the model, since this only impacts some of the participants, we tested whether there were significant differences in recruitment success between pregnant (41.5% success) and not obviously pregnant (33.3%) women. Since we found no significant difference in recruitment success [*t*(270.791) = 1.463, *p* = 0.145, correcting for significantly unequal variances], we collapsed across pregnancy status for the main analysis. Recruiter gender could not be evaluated because only one male recruiter participated. Recruiter age was not evaluated because there was very little diversity in age, with all recruiters falling within the range of 20–30 years old.

The results from the model indicate that only three of the hypothesized variables impacted recruitment success: if other recruiters were busy during the recruitment attempt (busy-ness), the script the recruiter used (script), and the recruiter’s race (see Table 1, Analysis 1). Since we had originally hypothesized that participant factors would be more important than qualities of the recruiter, we had not included any interactions between recruiter-driven factors in the original model. Consequently, to probe for interactions between recruiter race, busy-ness, and script, we re-ran the analysis including all of the variables of interest and their interactions. This changed only one result: the script the recruiter used was no longer significant by itself. Instead, there was a significant script by recruiter race interaction (see Table 1, Analysis 2).

**TABLE 1:** **Regression analyses predicting recruitment success in study 1**.

Regression models predicting recruitment success in study 1							
Analysis	Variable	*B*	SE	95% CI for odds ratio			*p*-Value
				Lower	Odds ratio	Upper	
Analysis 1 (without the interactions)	Script	0.171	0.077	1.022	1.187	1.379	0.025
	Ethnicity	0.668	0.276	1.134	1.950	3.351	0.016
	Busy-ness	0.551	0.225	0.370	0.576	0.897	0.015
Analysis 2 (with the interactions)	Recruiter race	1.264	0.436	0.120	0.282	0.664	0.004
	Busy-ness	0.563	0.225	0.366	0.569	0.885	0.012
	Script × recruiter race	0.280	0.123	1.040	1.323	1.683	0.023

Recruitment attempts were less likely to succeed when there were not other potential participants already engaged with a recruiter. The success rate when other recruiters were busy with potential participants jumped from 37.6 to 44.1%. Much of the success that occurred when other recruiters were busy stemmed from potential participants choosing these times to approach the recruiters themselves. Potential participants approached a recruiter 28 times across all observation periods. These were very likely to be successful, with an 85.7% success rate. The majority of participant approaches occurred when the other recruiter was busy (*n* = 20), as compared to when they were not busy (*n* = 8).

A recruitment attempt was less likely to be successful if the recruiter was non-White (29.0% success) as compared to if the recruiter was White (40.7% success) because non-White recruiters were more likely to be using scripts with lower success rates. The most successful scripts were directive, invoking either a University affiliation (37.6% success) or science (30.4% success). These were also very popular scripts, being used in 221 (62.1%) and 46 (12.9%) of interactions, respectively. The least successful script ways of approaching the participant were to simply say “Hello” (10.6% success) or to offer the parent or child a toy (0% success). Despite its lackluster success rate, “Hello” was surprisingly popular and was used in 47 (13.2%) of recruitment attempts. The usage rate of “Hello” was comparable to invoking science, despite the nearly 20% disparity in success rate between the two.

White recruiters used recruitment scripts that were more successful more often. For example, White recruiters invoked a University affiliation in 71.4% of observed recruitment attempts, while non-White recruiters used this script in 41.0% of attempts. By contrast, “Hello” was used by White recruiters 10.5% of the time and by non-White recruiters 24.8% of the time. Success rates with these scripts also differed. White recruiters were more successful with the University affiliation script (40.4% success) as compared to non-White recruiters using the same script (23.7% success). Conversely, non-White recruiters were slightly more successful with “Hello” (12.5% success) than were White recruiters (8.7%), but this was still not a very successful strategy for any group. It appears that non-White recruiters were using recruitment scripts that were less successful. Consequently, non-White recruiters’ success rate was lower than that of White recruiters.

The other variables we had hypothesized would influence the outcome of the recruitment attempt did not reach statistical significance. Recruitment success with White and non-White participants was nearly equivalent (37.1 and 38.0%, respectively). Females agreed to participate 36.6% of the time whereas males agreed 44.0% of the time. The number of people in the group accompanying the potential participant also did not significantly impact recruitment success, with success rates amongst people unaccompanied, with one companion, or with two or more companions being 41.7, 36.9, and 36.6% success, respectively.

### Discussion

Study 1 determined which factors may be influencing potential participants’ decisions. Contrary to our hypotheses, characteristics of the potential participant did not significantly predict recruitment success. The level of interest of other potential parents, recruiter race, and relatedly, the recruitment script did significantly predict the outcome. These results suggest two strategies to improve recruitment success. (1) That recruiters were more successful when co-recruiters are busy suggests that success in attracting parents begets recruitment success; the best time at which to recruit most heavily would be when co-recruiters are engaged with a family. (2) Using only the most successful recruitment scripts will allow recruiters to optimize their recruitment effort. The poor performance of “Hello,” as compared to invoking a University affiliation or science, suggests that using more directive pitches may result in more parents agreeing to participate, which is in keeping with previous research that has found that understanding participants’ informational needs and providing them with sufficient information to appreciate what they are being asked is important in increasing the likelihood of recruitment success (Young and Dombrowski, 1990). This may be particularly true with less-experienced recruiters. In this study, our non-White recruiters were less experienced than our White recruiters and we conjecture that this difference in experience, not race *per se*, resulted in non-White recruiters’ increased use of less successful recruitment scripts and lower levels of recruitment success. Randomly assigning only successful recruitment scripts to recruiters will allow recruiters to optimize recruitment success and will allow our analysis to determine whether recruiter race, over and above the effect of experience, impacts recruitment outcome.

Additionally, since study 1 only documented qualities of potential participants approached by the recruiter but not those of all potential participants who passed by the recruitment booth (i.e., the broader population available to the recruiter), it provided no information about whether the recruiters were targeting an unrepresentative or restricted sample. It also did not consider overall sampling rate (i.e., how many people were approached vs. the number that were present but not approached). Study 2 addresses these issues while attempting to increase diversity and success.

## Study 2: Applying the Lessons from Study 1 to the Next Large-Scale Recruitment Event

The second study was designed to determine whether we could use the lessons learned in study 1 to increase the number of families we recruited and our success with non-White families. We modified our procedure in two ways. First, once per hour, we randomly assigned scripts to recruiters, using only the scripts that were most successful in study 1. Experimental manipulation of the recruitment script was implemented to control for any recruiter-specific factors related to recruitment success (e.g., novice recruiters selecting less successful scripts). Second, we encouraged recruiters to recruit more heavily when other recruiters are engaged with potential participants. More vigorous recruitment during these times was expected to leverage other parents’ interest to encourage participation. We hypothesized that both of these strategies would increase our success rate.

To provide a baseline, we observed the characteristics of the people present and available for recruitment (i.e., walking past the recruitment booth), regardless of whether a recruiter approached them. We hypothesized that the population from which we were recruiting would reflect the larger metropolitan area from which it was drawn. We also hypothesized that the recruiters would not show differential rates of recruitment attempts of White and non-White potential participants.

To assess the reliability of the observational data, this second study also included a measure of inter-rater reliability. A second researcher independently recorded their observations for 25% of recruitment interactions and 25% of baseline observation periods. These observations were used to determine inter-rater reliability. Only the data from the primary observer were used in the analysis.

### Method

This study took place at a Developmental Studies recruitment booth at the 2012 Winter BabyTime Show, which occurred 6 months after the recruitment event at which study 1 took place. Successful recruitment again was defined as recruiters obtaining participants’ contact information.

#### Participants

Fifteen recruiters, six non-White (one Middle Eastern, one East Asian, one Pakistani, one Hispanic, one Sri Lankan, and one South-East Asian) and nine White, ranging in age from 19 to 30 years old (*M* = 22.2, SD = 2.886, range: 19–30 years), participated in the recruitment event. All recruiters provided informed consent to participate in the study and, consequently, were not blind to the aims of the study. This research was approved by the Ryerson University Research Ethics Board.

#### Procedure

As in study 1, a single observer observed and noted all interactions between recruiters and potential participants for 10 min every hour for all three 8-h days for a total of 24 observation periods. As before, they noted characteristics of the recruiter (age, gender, and ethnicity), characteristics of the potential participant (gender, White or non-White, obviously pregnant or not obviously pregnant, and number of people in their group), and characteristics of the interaction (were co-recruiters busy and what script was used).

**FIGURE 1:**
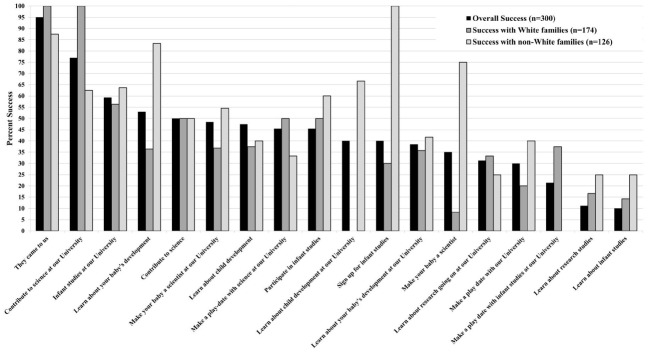
**Success rates of recruitment scripts overall and with White and non-White families**.

Every hour, each recruiter would randomly draw 1 of 15 recruitment scripts, written on a slip of paper, out of a hat. All recruitment attempts began with “Can I tell you a little bit about [how to/how you can]” and terminated with the randomly assigned script (see Figure 1 for all scripts). Once all of the scripts had been selected, an identical set of scripts was used to fill the hat, to ensure that the frequency with which scripts were assigned was approximately equal. Recruiters were asked to use only the one, randomly-selected script during the recruitment observation period. Recruiters were informed that they could use any other script outside of the observation period, although none did; recruiters used the randomly-assigned recruitment script throughout the hour, until they drew a new script.

In addition to observing recruitment attempts, the observer would spend an additional 10 min per hour observing the people who walked past the recruiters. During these baseline periods, observer(s) would document the following for each person who walked past the recruiter(s): gender, ethnicity (White or non-White), pregnancy status, group qualities, and whether a recruiter attempted to engage them. These periods provided a baseline for which individuals recruiters were attempting to recruit, to determine whether the characteristics of people to whom the recruiters spoke was comparable to the characteristics of people to whom the recruiters did not speak. During these baseline observation periods, the observer did not note the scripts being used or the outcome of the interactions.

On the baseline measures of whether the recruiter engaged the potential participant and participant age, gender, and race, we calculated Cronbach’s alpha as a measure of inter-rater reliability on 784 observations, representing six observation periods and 25% of all baseline observations. Reliability was high for the five variables evaluated: whether the recruiter engaged the potential participant (Cronbach’s α = 0.997), pregnancy status (Cronbach’s α = 0.996), gender (Cronbach’s α = 0.989), race (Cronbach’s α = 0.963), and number in the group (Cronbach’s α = 0.860).

For observations of recruitment, we calculated inter-rater reliability on 70 observed recruitment events, representing six observation periods and 25% of all recruitment observations. As for baseline measures, we calculated Cronbach’s alpha as a measure of inter-rater reliability. Reliability was high for all six variables evaluated: whether the recruiter engaged the potential participant (Cronbach’s α = 0.998), pregnancy status (Cronbach’s α = 0.974), gender (Cronbach’s α = 0.995), race (Cronbach’s α = 0.986), number in the group (Cronbach’s α = 0.962), and the outcome of the interaction (Cronbach’s α = 0.987).

### Results

The goal of the second study was to determine whether we could improve our recruitment success rate by implementing two changes: random assignment of recruitment scripts and more vigorous recruitment during periods when co-recruiters were busy speaking to other potential participants. Over the 3-day event, a total of 2657 potential participants were observed, 1868 during baseline observation periods and 789 during the 359 recruitment interactions observed. Of the 359 observed recruitment attempts, 38.1% (*n* = 137) were successful, 45.5% were unsuccessful (*n* = 163), and 16.4% (*n* = 59) were ineligible or did not clearly accept or decline. Excluding the ineligible participants, recruitment success rate was 45.7%. A total of 567 families were recruited. Our total success over our previous 2-year pre-intervention average was increased by 15.3%, which translated into 91 more participant families [*t*(3) = 13.135, *p* = 0.048]. Our success rate also significantly increased, from 37.6% at the previous show to 45.7% at this show [*t*(628.975) = 2.084, *p* = 0.038].

We observed 359 interactions during periods when we were documenting what the recruiter said and the outcome of the interaction. Recruiters attempted to recruit females most of the time (86.1%) and males less often (13.9%), which was not significantly different than the ratio of males to females engaged in study 1, *t*(766) = 0.094, *p* = 0.925. There was near parity in the frequency with which recruiters engaged not obviously pregnant (48.5%) and obviously pregnant (51.5%) females, which was significantly different from study 1 where only 42.5% of females were obviously pregnant, *t*(649.457) = 2.227, *p* = 0.026. As in study 1, because there was not a significant difference in recruitment success between pregnant (43.3% success) and not obviously pregnant women (46.0% success), *t*(258) = 0.444, *p* = 0.657, we collapsed across pregnancy status within females for our analysis of recruitment success.

Recruiters engaged non-White potential participants 40.1% of the time and White potential participants 59.9% of the time. This proportion of White to non-White is not significantly different than proportion of visible minorities in the metropolitan area (42.9% visible minorities, Statistics Canada, 2007), *t*(358) = 1.077, *p* = 0.282. This was also not significantly different from the proportion of White and non-White potential participants engaged in study 1, *t*(766) = 0.004, *p* = 0.997. The potential participant was alone 15.1% of the time, with one other person 56.4% of the time, with two people 23.7% of the time, and with three or more people 4.8% of the time. The majority of recruitment attempts occurred when co-recruiters were busy (52.9%) as compared to when they were not. This was not significantly different than the rate of recruitment during busy times in study 1, *t*(766) = 0.437, *p* = 0.662, suggesting that recruiters may not have recruited with more vigor during study 2’s busy times.

As in study 1, we performed a binary logistic regression, using the forward likelihood ratio to determine which variables were predictive of outcome and would be retained in the model. We had hypothesized that the predictive variables would change, consequent to our intervention. We anticipated that characteristics of the recruiter would no longer be significantly predictive of outcome, but the script used would remain predictive of outcome. We further expected that being busy, in that the co-recruiter was already engaged with a parent, would also be predictive of outcome, with busier times being more successful. Based on study 1, we did not anticipate any potential participant characteristics to predict recruitment success. Since previous literature suggests non-White participants volunteer less often, conflicting with the findings of study 1, we still included characteristics of the potential participant in our model. As in study 1, our analysis included only participants who were eligible and clearly accepted or declined (*n* = 300).

As in the previous study, three factors predicted the outcome of the model. Unlike in study 1, the race of the recruiter was not predictive of outcome. White and non-White recruiters were equally successful at this recruitment event [44.8 and 46.5%, respectively, *t*(298) = 0.281, *p* = 0.776]. Whether co-recruiters were busy was also not significantly predictive of recruitment outcome and was not retained in the model, despite significantly more success when the recruiters were busy (52.7%) as compared to when they were not (38.9%), *t*(297.903) = 2.127, *p* = 0.034. In study 2, the predictive factors were the script used, the race of the potential participant, and an interaction between the race of the potential participant and the script used (see Table 2).

**TABLE 2:** **Regression models predicting recruitment success in study 2**.

Regression models predicting recruitment success in study 2						
Variable	*B*	SE	95% CI for odds ratio			*p*-Value
			Lower	Odds ratio	Upper	
Script	0.106	0.030	1.112	1.048	1.179	0.005
Potential participant race	1.422	0.509	4.144	1.527	11.247	0.045
Script × potential participant race	0.089	0.045	0.915	0.838	0.998	<0.001

Race of the participant was predictive of success in the opposite direction than that predicted: recruitment success was higher with non-White than with White participants. More than half of non-White participants approached by a recruiter chose to participate (53.2%) whereas only 40.2% of White participants chose to participate. As compared to study 1, recruitment success with non-White potential participants increased significantly, *t*(262.868) = 2.709, *p* = 0.007. There was no significant difference between studies 1 and 2 in successful recruitment of White potential participants, *t*(377) = 0.433, *p* = 0.665.

The script was predictive of recruitment outcome. In study 2, recruiters were assigned 1 of 15 scripts. Although all were versions of successful scripts used in study 1, not all were equally successful. As illustrated in Figure 1, invoking a University affiliation was very successful. The top two scripts were: “contribute to science at our University” (76.9% success rate), and “infant studies at our University” (59.3% success rate). These success rates can be compared to similar scripts that did not mention a University affiliation. For example, the success rate for [can I tell you a little bit about] “infant studies” was only 10.0%; adding a University affiliation boosted success nearly sixfold to 59.3%. On the least successful end of the scale were scripts with impoverished information. The two least successful scripts were [can I tell you a little bit about] “infant studies” (10.0%) and [can I tell you a little bit about] “research studies” (11.1%), neither of which offered details about what the parent was being asked to do. Other small changes in wording also seemed to have an effect. For example, asking parents to contribute was generally better received than asking them to participate or sign up: “contribute to science” was 50.0% successful, whereas “participate in infant studies” and “sign up for infant studies” were only 45.5 and 40.0% successful, respectively.

Success rate interacted with race. White and non-White potential participants seemed to respond differently to certain scripts. The top three scripts for non-White potential participants were “sign up for infant studies” (100%), “learn about your baby’s development” (83.3%), and “make your baby a scientist” (75.0%). With White potential participants, these were 30.0, 36.4, and 8.3% successful, respectively (see Figure 1). The top three scripts for White potential participants were “contribute to science at our University” (100%), “infant studies at our University” (56.3%), and a three-way tie amongst “contribute to science,” “participate in infant studies,” and “make a play-date with science at our University” (each with a 50.0% success rate; see Figure 1).

#### Baseline Measures

Observers noted 1868 adults who walked past the recruiters during the twenty-four 10-min baseline observation periods. Most potential participants were accompanied by one other person (51.2%). Some were alone (16.7%), with two other people (22.9%), or with three or more other people (9.2%). The ratio of White to non-White potential participants was 61.1 to 38.9%. This proportion of non-White potential participants is significantly less than the proportion of minorities in the metropolitan area [42.9%, *t*(1867) = 3.528, *p* < 0.001]. Most potential participants who walked past the recruiters were female (69.1%) and only 30.9% were male. Slightly less than half of the females were pregnant (48.1%). This disparity in gender and high number of pregnant women was expected, given that this recruitment event was taking place at a trade show for parents and parents-to-be.

The goal of the baseline measure was to ensure that we were not differentially recruiting one gender or ethnicity. Given the make-up of the sample population, there was no statistically significant difference in the proportion of participants recruiters approached based on observable qualities of the participant: recruiters approached 29.3% of males and 32.3% of females, *t*(1166.958) = 1.392, *p* = 0.164; and 33.9% of obviously pregnant and 30.1% of not obviously pregnant females, *t*(1270) = 1.176, *p* = 0.240; and 33.0% of non-White and 30.3% of White potential participants, *t*(1840) = 0.998, *p* = 0.318.

### Discussion

Results from study 2 reveal that targeted recruitment strategies can be leveraged to significantly increase recruitment success. Two small changes—utilizing only the scripts that were successful previously and encouraging more recruitment when other recruiters were engaged with parents—boosted success by 15.3%, which translated into 91 more families across a 3-day recruitment event. Non-White families signed up proportionally more often than did White families. This greater success with non-White participants occurred despite the fact there were significantly fewer of them at our recruitment location, given the demographics of the city. This suggests that empirically evaluating recruitment strategies and using this information in future recruitment is a powerful way to increase inclusivity and success.

The key strategy that consistently predicted success, through an interaction with recruiter race in study 1 and through an interaction with participant race in study 2, was the script used by recruiters. What the recruiter said to engage the participant significantly impacted the likelihood that potential participants would sign up. Recruiter factors and participant factors (other than race) did not predict the outcome of a recruitment attempt.

## General Discussion

Recruitment is key to success in research using human participants. It ultimately determines the sample, which either sinks or buoys the study’s generalizability to the population of interest. We were successful in our main goal of increasing recruitment success and diversity by determining which factors predicted recruitment success within a typical recruitment interaction and leveraging these factors to recruit more non-White families. We found that what we say to potential participants and when we engage them predicts whether they are likely to accept our invitation to participate. In study 2, we manipulated what recruiters said to participants by randomly assigning only successful recruitment scripts. This intervention significantly improved recruitment success rate and the overall number of families recruited. With only successful scripts being used, we found that success rate differed between White and non-White potential participants across the scripts.

Within the literature, there seem to be three reasons offered as an explanation for the disparity in participation between White and non-White individuals. First, it has been suggested that researchers do not prioritize recruitment. Recruitment is often left to inexperienced junior researchers or research assistants (Patel et al., 2003). Previous research has found that recruiter experience is likely to increase recruitment success amongst hard-to-recruit groups (Yancey et al., 2006), potentially through crafting informed strategies (Quintana et al., 2006). High recruiter motivation and dedication (Fletcher and Hunter, 2003) and involvement of senior-level researchers in recruitment events (Patel et al., 2003) have also been suggested to be important, although these factors have not been empirically examined. Unfortunately, senior-level researchers do not typically involve themselves in recruitment (Patel et al., 2003). A related reason that has been offered as an explanation for why there is a disparity in participation of White vs. non-White individuals is that researchers view recruitment of a highly diverse sample as not worth the effort required. Troublingly, a lack of success at recruiting minority, relative to White, participants has been used to justify a lack of sample diversity and not engaging in further attempts to recruit non-White participants (Clay et al., 2003). This odd logic of using failure to recruit a diverse sample to justify not changing the precise recruitment strategy that resulted in the lack of diversity has prompted a call for re-evaluation of recruitment methods to create representative samples (Clay et al., 2003).

The least constructive rationale proposed to explain lack of success recruiting non-White participants has been to blame non-White participants for our lack of success in engaging them. Identification of any group as problematic is, itself, problematic, particularly since lack of success may stem from the way in which science treats this group. For example, one explanation for the lack of diversity in samples is that historical abuses of minorities in research studies has resulted in a high level of distrust of science amongst non-White potential participants, leading to lower levels of participation (Winett et al., 1974; Swanson and Ward, 1995). There is evidence that trust influences the likelihood of participation. Studies have found that low levels of trust make it less likely that minority participants would be willing to participate in research (e.g., African Americans; Williams and Corbie-Smith, 2006). Research has demonstrated that trust issues can be ameliorated by better communication between the researcher and potential participant (Yancey et al., 2006). It is not the case that we cannot increase recruitment of individuals from groups that have traditionally been considered to be hard to recruit.

We view all three theories that attempt to explain why there is a lack of diversity in research samples as being representative of the same problem: a lack of understanding of recruitment. If recruitment is left to inexperienced, junior researchers, these recruiters are unlikely to be aware of the most successful recruitment strategies or most harmful pitfalls. Consequently, they may only achieve success with groups that are traditionally easy to recruit (e.g., a WEIRD sample). In the current research, our success rate with White individuals did not improve from study 1 to study 2, suggesting that White potential participants may be less sensitive to how they are asked to participate. This may make it appear that recruitment strategies are effective and the group for which recruitment was less successful is the problem. Consequently, researchers may assume that aspects of the recruitment interaction itself must not matter, and therefore choose to allocate fewer resources to recruitment, perpetuating the cycle and making it less and less likely that the sample will be representative.

Studies 1 and 2 point to three simple, expedient strategies by which we increased recruitment of our target population. The first was to use only effective scripts. Effective scripts in the current study provided the participant with information about what they were being asked and included a university affiliation, whereas ineffective scripts were non-informative (e.g., “Hello”) and/or kitschy (e.g., “Make a play date with infant studies”). The second was to assign effective scripts to recruiters, decreasing the probability that less experienced recruiters used less effective scripts. The third was to increase recruitment during busy times, since parents were more likely to come ask us about research when other parents appeared interested. While these strategies were effective with our population, they may not generalize to all contexts. In particular, the scripts that work best likely vary in different populations (e.g., if the university is not viewed favorably in a particular community, invoking a university affiliation might decrease rather than increase recruitment success).

Discovering what is and is not effective within a given population requires empirical investigation, however the research into what is helping or hindering recruitment can be conducted during typical recruitment efforts, as in the current studies. This requires little time and effort on the part of the researcher. Empirically-supported recruitment has the potential to increase overall participant rates and, importantly, to reduce inequities in participation in psychological research. Including underrepresented populations allows for a fuller picture of the psychological construct and for greater generalizability. Inclusive recruitment efforts that lead to greater inclusivity in research may also serve to increase overall trust and positive perception of psychological research. This in turn may facilitate future recruitment of a diverse sample. This is similar to the pseudo-insider strategy, whereby the recruiter establishes themselves within the community or population of interest (Sixsmith et al., 2003). The pseudo-insider allows the researcher a better understanding of the needs, concerns, and perceptions of the potential participant. These can then be used to develop strategies that meet the needs of participants. Our two studies attempt to achieve the same understanding of what factors are influencing participants’ response to recruitment.

Evaluating our recruitment methods empirically shows four clear advantages: (1) it provides a base from which to build a better appreciation of the needs of potential participants, (2) it allows us to select and use only the most successful methods, thereby increasing recruitment success, (3) it allows some understanding of the way in which the sample may deviate from the population, and (4) it increases sample diversity by allowing us to access participants who may have been less likely to sign up when approached with a less-ideal method.

### Conflict of Interest Statement

The authors declare that the research was conducted in the absence of any commercial or financial relationships that could be construed as a potential conflict of interest.
